# Miscellany

**Published:** 1887-09

**Authors:** 


### Miscellany.

Guaiac in Quinsy.—The use of guaiac in quinsy has met with such success, that the drug is regarded by many as almost a specific for the disease; more recently, attention has been called repeatedly to the surprisingly good effect of salicylate of soda in acute tonsillitis, and the theory has been maintained that tonsillitis is closely connected with rheumatism; hence the success of the use of guaiac and salicylate of soda, both of which seem to have a specific action in rheumatism. The value of salicylic compounds in quinsy was first pointed out by Lennox Brown in 1878, in a work entitled, "The Throat and its Diseases," and at the same time he called attention to the relation between quinsy and rheumatism. The idea seems to be that because the salicylates are specifics for rheumatism themselves, whatever they cure must be a form of this disease, be it chorea, headache or sore throat. In a similar way it is argued that neuralgia, bronchitis, headache or whatever disease seems to yield to treatment by quinine, must be caused by malaria.—*Northwestern Lancet.*

Mamma in an Ovarian Tumor.—Von Velito claims to have discovered a mamma with glands and nipple complete in a dermoid ovarian tumor. This was hemispherical, and the size of the fist of a child. A nipple projected from it, which when pressed yielded two or three drops of a thick milky fluid. When examined under the microscope this showed numerous spherical fat globules and colostrum crystals floating in a milk-like fluid. The areola was of a pink color and surrounded by small pimples, from each of which projected a ¾ centimetres long fair hair. Microscopic sections of the mamma showed glandular tissue of similar nature to that met with in the normal breast. —*London Medical Record.*

---

Effects of Tobacco upon the Health.—Madame Walitzkaja, a Russian physician, has examined more than 1000 men, women and children employed in tobacco factories in Charcow. She finds that the constant exposure to the tobacco dust induces nervous disorders of a marked character, such as dilatation of the pupils, exaggeration of the tendo reflex, tremor and dyspnœa. The employees are also subject to headache, fainting, gastralgia, muscular spasms and nervous coughs, without any perceptible disease of air passages. The author has made experiments on rabbits and dogs by keeping them in an atmosphere containing tobacco dust and finds that similar effects are produced.—*Bull. Gen. de Therap.*

---

A writer in Vratsch believes that the posture in sleep influences the extension of a bronchitis. He found, for example, that in the 235 cases referred to, all of whom had this disorder, in ninety-seven it was left-sided, in seventy-two right-sided, and in sixty-six on both sides. He thinks that the preponderance of the bronchitis on the left side was due to the fact that there was a greater expansion of this side during sleep, and, consequently, a greater ingress of cold air or of the morbific particles causing the disease.—*Record.*

---

Several cases of priapism have been reported lately where all the ordinary forms of treatment failed to give relief, but the administration of large doses of iodide of potassium caused the gradual disappearance of the affection.

GASEOUS.—An unnecessary fuss is made over the gas-cure for consumption. Quite a number of physicians have been known to depend mainly upon gas for success in various branches of practice.—*Ex.*

---

SKIN DISEASES.—The most itching skin diseases may be relieved by a warm bath containing a handful of borax, and a like amount of bicarbonate of soda, in about thirty gallons of water.—*Ex.*

---

EQUAL parts of burnt alum and tannin sprinkled in powder upon venereal warts will dessicate them, and they can be rubbed off in a few days.—*Canada Med. Record.*

---

A QUACK recommends smoking for the treatment of sciatica, because it is a well-known fact that smoke will cure hams.

---

THE faculty of the University of Pennsylvania have banished cigarettes from the college grounds.

---

REBONY recommends the syrup of the lacto-phosphate of lime for the cure of night-sweats of phthisis.

